# An Update on Coat Protein Complexes for Vesicle Formation in Plant Post-Golgi Trafficking

**DOI:** 10.3389/fpls.2022.826007

**Published:** 2022-02-23

**Authors:** Kai Ching Law, Ka Kit Chung, Xiaohong Zhuang

**Affiliations:** Centre for Cell and Developmental Biology, State Key Laboratory of Agrobiotechnology, School of Life Sciences, The Chinese University of Hong Kong, Hong Kong, Hong Kong SAR, China

**Keywords:** protein sorting, coated vesicle formation, adaptor protein complex, retromer, retriever

## Abstract

Endomembrane trafficking is an evolutionarily conserved process for all eukaryotic organisms. It is a fundamental and essential process for the transportation of proteins, lipids, or cellular metabolites. The aforementioned cellular components are sorted across multiple membrane-bounded organelles. In plant cells, the endomembrane mainly consists of the nuclear envelope, endoplasmic reticulum (ER), Golgi apparatus, trans-Golgi network or early endosome (TGN/EE), prevacuolar compartments or multivesicular bodies (PVCs/MVBs), and vacuole. Among them, Golgi apparatus and TGN represent two central sorting intermediates for cargo secretion and recycling from other compartments by anterograde or retrograde trafficking. Several protein sorting machineries have been identified to function in these pathways for cargo recognition and vesicle assembly. Exciting progress has been made in recent years to provide novel insights into the sorting complexes and also the underlying sorting mechanisms in plants. Here, we will highlight the recent findings for the adaptor protein (AP) complexes, retromer, and retriever complexes, and also their functions in the related coated vesicle formation in post-Golgi trafficking.

## Introduction

Protein trafficking is a complex of tightly regulated physiological processes in plant. The fundamental principle of protein secretion is after a protein is synthesized in the endoplasmic reticulum (ER), it will be transported within the endomembrane system and finally reach its destiny to perform the corresponding cellular functions there. Proteins that synthesized at the ER are either retained or transported to post-Golgi compartments. It is determined by the properties of the protein, including conformation, amino acid-based retention signal, and the presence of transmembrane domain (Banfield, 2011). After modification, secretory proteins are transported following a cis-to-trans direction and eventually packaged into vesicles, which subsequently bud off from trans-Golgi cisternae. The formation of the budding vesicles is achieved by distorting the membrane conformation of the Golgi apparatus. The cytosolic face of the transport vesicles is coated with proteins, which are responsible for structural maintenance and cargo recognition of the vesicle.

**FIGURE 1:**
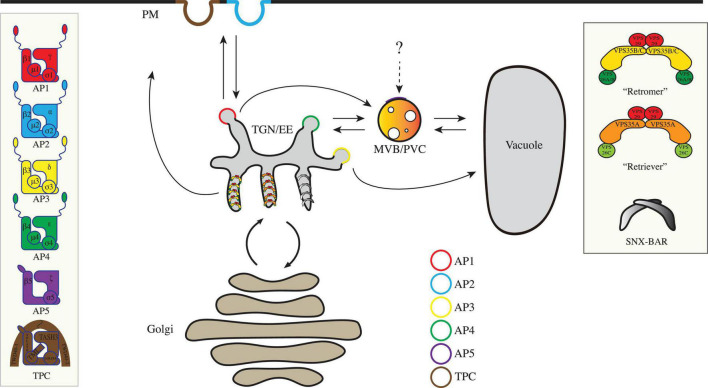
An overview of the coat protein complexes in plant post-Golgi trafficking. Core subunits for the coated vesicle machineries, including AP complexes (AP1-5), TPC, “retromer,” “retriever,” and SNX-BAR complexes in post-Golgi anterograde and retrograde transports are illustrated in boxes. AP, adaptor protein; MVB/PVC, multivesicular body/prevacuolar compartment; PM, plasma membrane; SNX, sorting nexin; TGN/EE, trans-Golgi network/early endosome; TPC, TPLATE complex; VPS, vacuolar protein sorting.

The anterograde transport (also known as forward transport) under the secretory pathway starts at the ER. Conventionally, after translation, proteins with transmembrane domains or signal peptides are targeted into the ER. Afterward, these proteins are transported to Golgi through the coat protein complex II (COPII) machinery for further modifications (Brandizzi and Barlowe, 2013). The Golgi apparatus is separated into three functionally distinct cisternae: the cis-Golgi, medial-Golgi, and trans-Golgi. These compartments are categorized by their corresponding resident proteins. The cis face of the Golgi is closest to the ER (Figure 1). From the trans face of the Golgi apparatus, various types of budding vesicles are formed and transported *via* the trans-Golgi network or early endosome (TGN/EE) to the plasma membrane (PM), apoplast, vacuole, or for cell plate formation (Figure 1), which requires a set of adaptor protein (AP) complexes localized on different subdomains.

On the other hand, some proteins can be transported back to the Golgi apparatus or TGN from PM or endosomes, instead of being sent into the lytic vacuole for degradation, which is also known as retrograde transport. The retromer complex was first identified in the yeast *Saccharomyces cerevisiae* and was shown to mediate endosome-to-Golgi retrieval of the carboxypeptidase Y (CPY) receptor Vps10p (Seaman et al., 1998). Later, conserved retromer complex was also found in other species, including mammals and plants. Recently, another complex named retriever has been discovered to function in retrograde trafficking in a retromer-independent manner (Mcnally et al., 2017). Retromer- or retriever-mediated protein transport is considered to be independent of coat assembly. However, recent structural studies in animal system have revealed the retromer and retriever assemble into arch-like coat to aid tubular vesicle formation from the endosomes (Leneva et al., 2021). In this article, we will update the recent discoveries for the functions of these complexes in protein sorting and vesicle formation for plant post-Golgi trafficking.

### Multiple Adaptor Protein Complexes, Same Coat?

Adaptor proteins are evolutionarily conserved among yeast, mammals, and plants (Arora and Van Damme, 2021). APs are divided into five complexes (AP1–5), which function in different localizations. Except the poorly understood AP5, other APs in plants have been found to form heterotetrametric complexes, which consisting of two large adaptin subunits (γ1/2 and β1/2 for AP1, α and β1/2 for AP2, δ and β3 for AP3, and ε and β4 for AP4), a medium subunit (μ1–4), and a small subunit (σ1–4) (Figure 1 and Table 1). Regarding AP5, it has been shown that it consists two large subunits (β5 and ζ) and a medium subunit (μ5); yet, the presence of σ subunit is still not clear in *Arabidopsis* (Hirst et al., 2011).

**TABLE 1:** A list of the core coat protein complexes in plant post-Golgi trafficking.

Coat protein complex	*H. sapiens*	*S. cerevisiae*	*A. thaliana*	Accessory Proteins	Known Cargo Motif	Pathway
AP1	γ1, 2	γ1	γ1, 2	EPSIN,	?	TGN/EE to PM
	β1, 2	β1, 2	β1, 2	GGAs	?	
	μ1A, B	μ1	μ1, 2		YXXØ*	
	σ1A, B, C	σ1	σ1, 2		[D/E]XXXL[L/I]	

AP2	αA, C	α	α1, 2	CALM,	?	PM to TGN/EE
	β1, 2	β1, 2	β1, 2	epsin,	?	
	μ2	μ2	μ2	ARH,	YXXØ*	
	σ2	σ2	σ2	β-arrestin	[D/E]XXXL[L/I]	

AP3	δ	δ	δ	?	?	EE to vacuole/lysosome, TGN/EE to vacuole/lysosome
	β3A, B	β3	β3		?	
	μ3A, B	μ3	μ3		YXXØ*	
	σ3A, B	σ3	σ3		[D/E]XXXL[L/I]	

AP4	ε	ε	ε	Tepsin,	?	TGN to vacuole, TGN to specialized compartments
	β4	β4	β4	MTV	?	
	μ4	μ4	μ4		YXXØ*	
	σ4	σ4	σ4		[D/E]XXXL[L/I]	

AP5	ζ	ζ	ζ	SPG11/15	?	late endosome to TGN, ?
	β5	β5	β5			
	μ5	μ5	μ5			
	σ5	σ5	/			

TPC	/	/	TSPOON (LOLITA)	CALM,	?	PM to TGN/EE
	/	/	TSAUCER (TASH3)	Epsin,		
	/	/	TCUP (TML)	ARH,		
	/	/	TPLATE	β-arrestin		
	/	/	TTRAY1,TTRAY2 (TWD40-1, TWD40-2)			
	/	/	AtEH1(Pan1), AtEH12(Pan1)			

Retromer	VPS26	Vps26p	VPS26A, VPS26B	SNX3,	ØX[L/M/V]; [ILMV]x[FY]xx2-13ØxØ or [FYW]x[FY]x3-15ØxØ; [-][-]x[-][ST]xØ	Vacuole/lysosome to Endosome, Endosome to Golgi/PM
	VPS29	Vps29p	VPS29	SNX27,		
	VPS35	Vps26p	VPS35B, VPS35C	WASH		

Retriever	VPS26c	/	VPS26C	SNX17,	NPxY/NxxY	
	VPS29	/	VPS29	CCC complex,		
	VPS35L	/	VPS35A	WASH		

SNX-BAR	SNX1	Vps5p	SNX1	SNX5/6,	[ILMV]x[FY]xx2-13ØxØ or [FYW]x[FY]x3-15ØxØ; [-][-]x[-][ST]xØ	
	SNX2		SNX2A,SNX2B	WASH		

*Ø represent hydrophobic amino acid, and X represent any amino acid.

Among all the APs, AP2 is one of the most well-categorized complexes in mediating clathrin-mediated endocytosis (CME) from the PM to endosomes (Figure 1; Jackson et al., 2010). The clathrin coat in *Arabidopsis*, which has been extensively reviewed, forms a triskelion shape that consists of three heavy chains and three light (Paul and Frigerio, 2007). Generally, the assembly of CCV is divided into several steps: recruitment of AP complexes, cargo selection, coat nucleation, and vesicle budding.

In animal model, it is suggested that only AP1 and AP2, but neither AP3 nor AP4, are clathrin-dependent (Hirst et al., 1999). However, recent findings showed that AP3 may also interact with clathrin (Kural et al., 2012; Zelazny et al., 2013). APs recognize and interact with multiple motifs that can be identified on their cargos. The tyrosine (YXXØ; Y, tyrosine residue; X, any amino acid; and Ø, an amino acid with a bulky hydrophobic side chain) and dileucine ([D/E]XXXL[L/I]; D, aspartic acid; E, glutamic acid residue; and L, leucine residue) motifs are the most well-documented (BONIFACINO and Traub, 2003; Mattera et al., 2011). Tyrosine motif has a binding affinity toward μ subunit of APs, whereas dileucine motifs are recognized by σ subunit of APs. Different from other APs, AP5, which was identified in 2011 in Hela cell line, is localized to a late endosomal compartment for retrograde transport to Golgi (Hirst et al., 2011). Notably, a novel complex, named TPLATE complex (TPC), was recently identified to mediate AP2-independent CME, which however seems lost in animals and fungi (Gadeyne et al., 2014; Hirst et al., 2014). In the followings, we will further discuss the different functions of each AP subcomplex in plant developments.

### AP1

AP1 is localized at TGN, which plays an important role in lysosomal or vacuolar trafficking in yeast, mammals, and plants (Arora and Van Damme, 2021). There are two isoforms of AP1 μ subunit (AP1 μ1 and AP1 μ2), which are localized at TGN, and double-knockout of two AP1 μ subunits is nearly pollen-lethal (Park et al., 2013). Loss-of-function of AP1 μ1 adaptin leads to pleiotropic growth defects in *Arabidopsis*, and auxin signaling is compromised due to asymmetric localization of PIN-FORMED2 (PIN2) auxin transporter (Wang et al., 2013). In addition, it is also reported that the formation of cell plate requires AP1-dependent vesicle transport during cytokinesis (Teh et al., 2013). Particularly, AP1 is also involved in cargo recycling from endosomes in yeast, mammals, and plants (Robinson, 2004; Zysnarski et al., 2019). For example, it has been shown that AP1 μ subunit deficiency suppresses the recycling of brassinosteroid insensitive1 (BRI1), a key receptor for the plant hormone brassinosteroid, from Brefeldin A (BFA) compartments to the PM (Wang et al., 2013). The dileucine sorting motif serves as one main sorting signal recognized by AP1, which is demonstrated by studying vacuolar targeting of *Arabidopsis* vacuolar ion transporter1 (VIT1), showing that knockdown of AP-1 γ subunit results in relocalization of VIT1 to the PM (Wang et al., 2014). Other types of sorting signals have also been reported for AP1. For example, plant vacuolar sorting receptor 4 (VSR4), which is required for the transportation of vacuole-localized protein from ER to vacuole, interacts with AP1 μ2 subunit through the tyrosine-sorting motif (Nishimura et al., 2016). Recently, it has been demonstrated that AP1 can physically interact with EPSIN1 for AP1 vesicle formation from a specific subdomain of TGN/EE (Heinze et al., 2020). This finding thus provides a novel insight into the role of accessory protein in AP1-dependent vesicle transport.

### AP2

AP2 localizes exclusively at the PM (Figure 1). During CME, AP2 is involved in nucleation, cargo selection, and clathrin coat assembly (Mcmahon and Boucrot, 2011). Similar to AP1, tyrosine and dileucine motifs are also recognized by AP2 during cargo selection (Fan et al., 2013). By far, AP2 has been implicated to function in various plant physiological processes. For example, AP2 mediates endocytosis of several hormone regulators, including PIN1/2 (auxin signaling) and BRI1 (brassinosteroid signaling) (Di Rubbo et al., 2013; Fan et al., 2013; Kim et al., 2013). Moreover, AP2 μ subunit is required for effector-triggered immunity (ETI) response (Hatsugai et al., 2016). Phenotypic analysis of homozygous *ap2* σ mutant shows dwarfism, altered vascular pattern, and multiple abnormalities in cotyledon development and organogenesis (Fan et al., 2013; Kim et al., 2013). Additionally, AP2-deficient mutant displays defects in floral organ development and reproduction (Kim et al., 2013; Yamaoka et al., 2013). Interestingly, depletion or inhibition of the AP1 adaptor subunit and clathrin recruitment to TGN also disturbs AP2-mediated CME, and vice versa, suggesting a crosstalk between AP2-dependent CME and AP1-dependent post-Golgi trafficking (Yan et al., 2021).

### AP3

AP3 was discovered by searching sequence analogy after the identification of AP1 and AP2. Each subunit of AP3 is encoded by a single copy of gene (Diane et al., 2008), but the function of AP3 in plants is still not well understood. AP3 has the ability to bind with clathrin, which however seems dispensable for AP3-coated vesicle formation. AP3 colocalizes with TGN markers in different eukaryotic systems (Feraru et al., 2010); yet, AP3-mediated trafficking bypasses the traditional prevacuolar compartment or multivesicular body (PVC/MVB)-vacuole route (Zwiewka et al., 2011). Genetic mutant screening showed that *protein affected trafficking2* (*pat2*) mutant, which was later been categorized as the β subunit of AP3, displays defects in vacuolar morphology and protein degradation (Feraru et al., 2010). Interestingly, *ap3* mutants (either β or μ subunit) show almost normal morphology under normal growth conditions (Feraru et al., 2010; Kansup et al., 2013). In addition, a recent study showed that AP-3 mediates the transport of PROTEIN S-ACYL TRANSFERASE10 (PAT10) and VAMP711 to tonoplast in a Rab5-independent manner (Feng et al., 2017). Of note, abnormal vacuole organization was also observed in *ap3* pollen cells, further supporting the role of AP3 in vacuolar trafficking for pollen tube growth (Feng et al., 2018).

### AP4

In *Arabidopsis*, each subunit of AP4 is encoded by a single copy of gene (Fuji et al., 2016). AP4 colocalizes with TGN marker SYNTAXIN OF PLANTS 43 (SYP43), but not with the μ subunit of AP1, implying that different subdomains of TGN are required for the localization of AP1 and AP4 (Fuji et al., 2016). The root lengths of *ap4ß* and *ap4*μ are significantly shorter than that in wild type; yet, double-knockout of these two subunits does not induce an additive effect (Müdsam et al., 2018). AP4 mutants are also hypersensitive to avirulent bacterial infection, probably due to defects in membrane fusion of vacuoles, suggesting that AP4-meidated protein trafficking plays additional role in plant immunity (Hatsugai et al., 2018). Moreover, mutagenesis experiment demonstrates that sorting of NATURAL RESISTANCE-ASSOCIATED MACROPHAGE PROTEIN 3 (NRAMP3) and NRAMP4 to vacuole requires interaction between AP4 and the dileucine motifs in NRAMP3/4 (Müdsam et al., 2018). Recently, MODIFIED TRANSPORT TO THE VACUOLE1 (MTV1), an EPSIN-like protein, which locates at TGN in mediating vacuolar transport, shows a strong binding affinity with AP4 than other AP complexes (Heinze et al., 2020). MTV1 has been previously discovered to function in clathrin-dependent vacuolar transport (Sauer et al., 2013). The MTV1/AP4 interaction defines a unique subdomain on TGN/EE, which is separated from the aforementioned EPSIN1/AP1 region. Recently, using super-resolution confocal live imaging microscopy, it has been nicely showed that AP1-mediated secretory and AP4-mediated vacuolar trafficking pathways are indeed initiated from distinct zones on TGN in *Arabidopsis*, further supporting that different cargo sorting subdomains exist on plant TGN (Shimizu et al., 2021).

### TPLATE Complex

Very recently, another novel AP complex termed TSET/TPC was reported, which consists of TSPOON (LOLITA), TSAUCER (TASH3), TCUP (TML), TPLATE, TTRAY1 (TWD40-1), and TTRAY2 (TWD40-2), and the counterpart in *Arabidopsis* plants involves additional two plant-specific subunits, named AtEH1/Pan1 and AtEH2/Pan1 (Gadeyne et al., 2014; Hirst et al., 2014; Figure 1). One of the TPC subunits, TPLATE, was first identified in *Arabidopsis* to participate in clathrin-mediated vesicle trafficking and cell plate formation (Van Damme et al., 2006). Later on, data from different organisms suggest that TPC in *Arabidopsis* plants is an octameric complex, whereas TSET is a hexametric complex in *Dictyostelium* (Gadeyne et al., 2014; Hirst et al., 2014). In plants, it was demonstrated that both TPC and AP2 participate in CME (Zhang et al., 2015). In a recent study, it has been shown that TPC is necessary for PM association of clathrin independent of AP2 (Wang et al., 2016). These results indicate that plant cells may initiate corresponding AP complexes for CME in response to different developmental signals. The molecular architectures of TPC are recently solved, revealing high similarities between TPC and the AP2-clathrin complex at the structural level (Yperman et al., 2021). In another study, a remarkable membrane-bending activity of TPC subunits during CCV formation was observed, which indicates a distinct mechanism of TPC in mediating plant endocytosis (Johnson et al., 2021). It would be interesting for future studies to further investigate how different signals are perceived by TPC- and the AP20-clathrin-mediated pathways.

### Retromer and Retriever: Non-classical Coats for Tubular Vesicle Formation

Once the proteins have accomplished their functions, some will be recycled back to the Golgi apparatus or TGN, from PM or endosomes through the retrograde trafficking to reduce energy expense. In addition to the classical coated vesicle formation, tubular vesicles containing retrograde cargoes are often observed on the edges of endosomes in animal cells (Chen et al., 2019). Different from the AP complex-mediated anterograde transport which requires extra structural proteins (e.g., clathrin) for creating membrane curvature, tubular vesicle formation is largely dependent on a different set of machineries which possess the ability to induce membrane remodeling and tubulation (Oliviusson et al., 2006). In tubular vesicle formation during retrograde transport, cargoes are recognized by their corresponding sorting nexin (SNX) partners. SNX proteins associate with the phosphatidylinositol 3-phosphate on the membrane and provide a platform for the rest of the machineries to attach to. The core retromer or retriever subunits are then recruited to the membrane surfaces to form a super complex. Subsequently, the super complexes form a chain extending along the endosome, which further induces membrane curvature and constructs a tubule-shape extension (Chen et al., 2019). Next, we will mainly discuss the functions of three core complexes, which include retromer, retriever, and SNX-BAR in retrograde transport from endosomes (Figure 1 and Table 1).

### Retromer

Originally, retromer system was discovered in yeast 24 years ago by screening mutants with the mislocalization of Vps10p and the abnormal excretion of CPY in yeast (Seaman et al., 1998). It is proposed that Vps26p, Vps29p, and Vps35p in yeast form a multimeric complex to maintain the correct localization of Vps10p and CPY with a vacuolar sorting function (Seaman et al., 1998). Later, this system was found to be conserved in most eukaryotes and extended further, rather than a simple vacuolar cargo sorting system to the vacuole (Oliviusson et al., 2006; Koumandou et al., 2011). Moreover, the retromer complex is responsible for recycling materials from endosome to PM, TGN, and Golgi (Chen et al., 2019).

In yeast, Vps26p, Vps29p, and Vps35p together form a trimeric core retromer, and lack of these subunits would result in abnormal endosomal morphology (Banta et al., 1988), abnormal protein transport (Steinfeld et al., 2021), and excretion of vacuolar enzyme (Seaman et al., 1998). In mammals, deficiency of VPS35 and VPS26 would lead to the dysfunction of hippocampal and neurodegeneration (Muhammad et al., 2008). It is suggested that suppression of retromer would cause an accumulation of soluble α5β1-integrin, a neurotoxic peptide, and subsequently result in the abnormality in neurons (Muhammad et al., 2008). Further studies have found that the absence of retromer would lead to a similar accumulation of other peptides related to neurodegeneration (Sullivan et al., 2011).

Key subunits in retromer complexes are also conserved in *Arabidopsis* plants. There are two VPS26 paralogues, three isoforms of VPS35, and one VPS29 in *Arabidopsis*, which have been long considered to function redundantly in the retromer complex. However, recently, it has been shown that VPS26A/B-VPS29-VPS35B/C might function as the core retromer complex, whereas the other subunits, VPS26C-VPS29-VPS35A, probably constitute the core retriever complex, which will be introduced later (Oliviusson et al., 2006; Mcnally et al., 2017). Up till now, our understanding of the core retromer complex is largely derived from genetic studies of the retromer subunits, which have unveiled the essential roles of different subunits in plant survival and development. VPS26A and VPS26B might function redundantly in the retromer complex, which is supported by the observation that the double-knockout mutant showed a severely compromised growth, whereas single-knockout of VPS26A or VPS26B would not cause any phenotype (Zelazny et al., 2013). VPS35B and VPS35C have also been suggested to play a redundant role in the core retromer. Two mutant lines have been identified for VPS35B, a total knockout line *vps35b-1* and a knockdown line *vps35b-2* (Yamazaki et al., 2008). Evident developmental defects, such as early leaf senescence and dwarfism, are only observed in the *vps35b-1 vps35c-1* double-mutant, whereas no significant growth defect has been observed in *vps35b-2 vps35c-1* double-mutant line, thus implying a dosage-dependent regulation role of VPS35B/C (Yamazaki et al., 2008). In retromer-deficient mutants, 12S seed storage proteins are secreted out and accumulate in the extracellular matrix. Meanwhile, protein storage vesicles show a defect with a reduction of size and increase on number (Yamazaki et al., 2008). Differently, with a single copy in *Arabidopsis* genome, depletion of VPS29 is more severe when compared to other retromer mutants. *vps29* mutant exhibits the dwarfism (Shimada et al., 2006) and abnormal cotyledons in shape and size (Jaillais et al., 2007). The core retromer subunits have been shown to participate in the sorting and recycling of PIN1, an important regulator for polar auxin transport in plants (Jaillais et al., 2007). As a result, defects including reduced gravity sensitivity as seen in *pin1* mutant are also observed in retromer mutant *vps29* (Jaillais et al., 2007).

In addition, SNX proteins have long been considered as an adaptor in retromer-mediated retrograde trafficking. Previous studies suggested that the cargo sorting specificity is mediated by the core retromer subunit, but recent studies showed that SNX partners also participate in cargo recognition and membrane binding (Leneva et al., 2021). In human, retromer complex cooperates with SNX3, SNX27, and SNX17 respectively for different cargoes (Mcnally et al., 2017; Healy et al., 2021). For example, retromer-SNX3 recognizes the cargo through a Øx(L/M/V) motif, whereas retromer-SNX27 binds to the PDZ domain in β2-adrenoreceptor (Lauffer et al., 2010). Differently, a NxxY motif in α5β1-integrin is recognized by retriever-SNX17 (Mcnally et al., 2017). However, some cargoes are also mediated by both retromer-SNX27 and retriever-SNX17 subcomplexes. Recently, a viral protein HPV-16 L2 has been reported to perform dual recruitment of retromer and retriever complexes simultaneously to assist the infection process, which provides a novel insight on how the two systems cooperate during infection (Pim et al., 2021). In *Arabidopsis thaliana*, counterparts for SNX3 have been recently reported, but whether they perform a similar function in plants is still unclear. In addition, no SNX27 and SNX17 homologs have been identified yet (Healy et al., 2021).

Besides SNX proteins, WASH complex is also reported as an accessory protein in retromer-dependent trafficking in animal cell. VPS35 would recruit WASH complex to the tubulation site through the direct interaction with FAM21, a subunit of WASH complex. The ability to recruit actin polymerization machineries for WASH complex would provide an additional mechanical force to facilitate the tethering of cargo from and out of the endosome (Jia et al., 2012). However, the WASH complex has not been discovered in plant and yeast (Jia et al., 2012; Wang et al., 2018).

### Retriever

Retriever was first discovered in 2017 by searching SNX17-interacting proteins using proteomic method (Mcnally et al., 2017). It was found that the recycling of α5β1-integrin on the cell surface from being degraded in the lysosome in mammalian cell requires the retriever subcomplex, but independent of the core retromer complex. Similar to retromer, retriever is a heterotrimeric complex composed of three subunits, namely VPS29, VPS35L, and VPS26C, which are conserved across most eukaryotes including plants, but absent in yeast (Mcnally et al., 2017). Meanwhile, more than 120 cell surface proteins were identified as retriever-dependent cargoes (Mcnally et al., 2017).

A counterpart of retriever complex in plants was also suggested very recently (Jha et al., 2018). It was shown that VPS26C interacts with VPS35A but not with VPS35B, and residents on BFA-insensitive compartments (Jha et al., 2018). Furthermore, loss-of-function analysis showed that deficiency of VPS26C, VPS29, or VPS35A affects root hair length after mannitol treatment, but no obvious changes were detected between wild type and other retromer single mutants (*vps26a, vps26b, vps35b*, and *vps35c*). This study provides the first evidence that VPS26C-VPS35A and VPS26/B-VPS35B/C might form distinct subcomplexes with differential functions in root length growth in *Arabidopsis*. Of note, a previous study also reported a possible divergent role of different retromer subunits in mediating PIN1 recycling (Nodzynski et al., 2013). Malfunction of VPS35a leads to a greater accumulation of PIN1-GFP aggregates and a stronger defect in vacuole morphology in *Arabidopsis* roots. Such defects were not evident when both *vps35b* and *vps35c* were knockout, although VPS35C has a higher expression in the root tissues. Therefore, further efforts are required to test whether VPS35A-VPS26C and VPS35B/C-VPS26A/B function in plant “retromer” or “retriever” subcomplex for the recycling of different cargos.

Another subcomplex called Commd/CCDC22/CCDC93 (CCC) complex also binds to SNX17 and retriever for the assembly of the commander complex. In mammals, it is found that CCC complex interacts with WASH complex on the endosomes to the recruitment of retriever to the endosomes (Bartuzi et al., 2016). Subunits of the CCC complex in *Arabidopsis* genome have also been identified, including coiled-coil domain-containing protein 22 (CCDC22) and coiled-coil domain-containing protein 93 (CCDC93). However, it awaits further study to reveal whether they function together with plant “retriever” in endosome sorting (Healy et al., 2021). Particularly, in human, commander complex also consists of 7 Commd proteins, but no homologs of these proteins can be found in *Arabidopsis thaliana* (Healy et al., 2021).

## SNX-BAR

The SNX-BAR subfamily has previous been considered as a part of retromer complex. SNX-BAR subfamily is featured with a structural BAR domain, and the SNX complex may utilize the BAR domain for endosome tubulation (Peter et al., 2004). However, accumulating evidence in both animals and plants suggested that SNX-BAR proteins themselves function independently of the core retromer complex, and there is still no evidence showing a interaction between SNX and retromer in plants yet (Nisar et al., 2010; Pourcher et al., 2010). A retromer-independent role of SNX-BAR is further supported by the evidence that CI-MRP (cation-independent mannose-6-phosphate receptor) requires SNX-BAR for its recycling, but retains its normal localization in retromer-depleted cells (Kvainickas et al., 2017). Similarly, it has also been suggested that retromer in plants might function independent of SNX proteins (Heucken and Ivanov, 2018), as triple mutant *snx1/snx2a/snx2b* only shows minor developmental defect (Pourcher et al., 2010), whereas *vps35a/vps35b/vps35c* mutation leads to a severe defect or even embryo lethal (Yamazaki et al., 2008). SNX1 either forms a homodimer or heterodimer with SNX2 for different cargoes. For example, SNX1-containing endosomes are specific for PIN2 recycling (Jaillais et al., 2006). In addition, SNX1 homodimer interacts with biogenesis of lysosome-related organelle complex subunits, BLOS1 and BLOS2, for their endosome to vacuole trafficking (Cui et al., 2010), whereas SNX1-SNX2B heterodimer regulates recycling of an iron transporter IRT1 in TGN (Ivanov et al., 2014).

It has also been reported that SNX-BAR proteins coordinate with CLASP, a microtubule-associated protein and FAB1 (formation of aploid and binucleate cells1), to link endosome with microtubule (Ambrose et al., 2013; Hirano et al., 2015). In both *clasp* and *fab1* mutants, SNX1-positive endosomes displayed an altered morphology, which support that microtubule is essential for SNX1 endosome formation. It is also noted that *clasp* null mutant only affects PIN2 transport as SNX1, but deficiency of FAB1 leads to mislocalization of both PIN1 and PIN2. Thus, FAB1 might perform additional function to regulate PIN proteins independent of SNX1 and CLASP.

## Summary

The cumulative knowledge of anterograde and retrograde trafficking in other eukaryotic systems has been made in recent years. Yet, the molecular mechanism for different sorting machineries in plants is still less well understood. Plants have evolved distinct trafficking machineries for sorting plant-specific proteins for different developmental processes. For example, the TPC has been demonstrated to participate in plant hormone regulation, cell wall proteins, or cell plate proteins trafficking (Van Damme et al., 2006; Wang et al., 2016). Moreover, AP1 and AP4 are both localized on TGN/EE, but very recently, with the advancement of high-resolution imaging technology, EPSIN1/AP1 and MTV1/AP4 subcomplexes were resolved to operate on distinct TGN/EE subdomains (Heinze et al., 2020). This raises out another question: how do AP1 and AP4 distinguish their specific cargoes on TGN/EE in two independent pathways or whether they function in parallel with overlapping cargos? In another finding using tandem mass spectrometry analysis, only AP1, AP2, and AP4 subunits were identified in enriched CCV proteome in Arabidopsis (Dahhan et al., 2021). It is possible that other accessory proteins might be involved to assemble as coatomer with similar function as the clathrin cage in AP3 vesicles. Therefore, searching the AP3 interactome would provide more insights for the assembly of AP3 vesicles.

In comparison with the well-characterized AP subcomplexes, our understanding for sorting mechanisms of the retromer and retriever complexes is still very limited. Many questions await further study: Whether retromer and retriever function redundantly or operate separately in plant systems? Are they distributed on the separate subdomains of endosomes and how they are recruited? What are the sorting motifs specific to retromer or retriever in plants? What are the plant cargoes mediated by retriever?

Understanding the trafficking system in cells would provide a new tool to engineer protein sorting and secretion pathway for future application. For example, by engineering the sorting mechanism, plant growth signals or cargoes can be properly sorted to achieve a higher growth rate and stress tolerance, or by sorting the important cargo proteins to plant storage vacuole, it would increase the yields and quality for the development of plant-based bioreactor. Further investigations into the sorting mechanism and corresponding cargoes in plants are certainly required, which would greatly pave the way for future applications.

## Author Contributions

XZ designed the concept of the manuscript. KL, KC, and XZ wrote and edited the manuscript. All authors contributed to the article and approved the submitted version.

## Conflict of Interest

The authors declare that the research was conducted in the absence of any commercial or financial relationships that could be construed as a potential conflict of interest.

## Publisher’s Note

All claims expressed in this article are solely those of the authors and do not necessarily represent those of their affiliated organizations, or those of the publisher, the editors and the reviewers. Any product that may be evaluated in this article, or claim that may be made by its manufacturer, is not guaranteed or endorsed by the publisher.
